# Comparison between synchronized and non-synchronized ventilation and between guided and non-guided chest compressions during resuscitation in a pediatric animal model after asphyxial cardiac arrest

**DOI:** 10.1371/journal.pone.0219660

**Published:** 2019-07-18

**Authors:** Gema Manrique, Miriam García, Sarah N. Fernández, Rafael González, María J. Solana, Jorge López, Javier Urbano, Jesús López-Herce

**Affiliations:** 1 Pediatric Intensive Care Department, Hospital Universitario Gregorio Marañon, Madrid, Spain; 2 Maternal and Child Public Health Department, Facultad de Medicina, Universidad Complutense de Madrid, Madrid, Spain; 3 Instituto de Investigación Sanitaria Gregorio Marañón, Hospital Universitario Gregorio Marañón, Madrid, Spain; 4 Research Network on Maternal and Child Health and Development (RedSAMID), Hospital Universitario Gregorio Marañón, Madrid, Spain; State University of New York at Buffalo, UNITED STATES

## Abstract

**Introduction:**

There are no studies comparing synchronized and non-synchronized ventilation with bag-valve mask ventilation (BVMV) during cardiopulmonary resuscitation (CPR) in pediatric patients. The main aim is to compare between synchronized and non-synchronized BVMV with chest compressions (CC), and between guided and non-guided CC with a real-time feedback-device in a pediatric animal model of asphyxial cardiac arrest (CA). The secondary aim is to analyze the quality of CC during resuscitation.

**Methods:**

60 piglets were randomized for CPR into four groups: Group A: guided-CC and synchronized ventilation; Group B: guided-CC and non-synchronized ventilation; Group C: non-guided CC and synchronized ventilation; Group D: non-guided CC and non-synchronized ventilation. Return of spontaneous circulation (ROSC), hemodynamic and respiratory parameters, and quality of CC were compared between all groups.

**Results:**

60 piglets were included. Twenty-six (46.5%) achieved ROSC: A (46.7%), B (66.7%), C (26.7%) and D (33.3%). Survival rates were higher in group B than in groups A+C+D (66.7% vs 35.6%, p = 0.035). ROSC was higher with guided-CC (A+B 56.7% vs C+D 30%, p = 0.037). Piglets receiving non-synchronized ventilation did not show different rates of ROSC than synchronized ventilation (B+D 50% vs A+C 36.7%, p = 0.297). Non-synchronized groups showed lower arterial pCO_2_ after 3 minutes of CPR than synchronized groups: 57 vs 71 mmHg, p = 0.019. No differences were found in arterial pH and pO_2_, mean arterial pressure (MAP) or cerebral blood flow between groups. Chest compressions were shallower in surviving than in non-surviving piglets (4.7 vs 5.1 cm, p = 0.047). There was a negative correlation between time without CC and MAP (r = -0.35, p = 0.038).

**Conclusions:**

The group receiving non-synchronized ventilation and guided-CC obtained significantly higher ROSC rates than the other modalities of resuscitation. Guided-CC achieved higher ROSC rates than non-guided CC. Non-synchronized ventilation was associated with better ventilation parameters, with no differences in hemodynamics or cerebral flow.

## Introduction

The main cause of pediatric cardiac arrest (CA) is respiratory failure leading to hypoxia and ischemia. Therefore, airway management and maintaining adequate ventilation are crucial during cardiopulmonary resuscitation (CPR) in children [1].

The 2015 ILCOR guidelines recommend a synchronized 15:2 compression-to-ventilation ratio during bag-valve-mask ventilation (BVMV) for pediatric CPR [2–4]. After tracheal intubation, a fixed respiratory rate of 10 to 12 bpm is recommended [3,5] while delivering continuous chest compressions (CC).

The recommendation to deliver synchronized insufflations with CC during BVMV relies on the theory that CC may interfere with ventilation in the absence of a secured, advanced airway [4]. Providing ventilation in asphyxial cardiac arrest has demonstrated to achieve better or equal outcomes in both human and animal studies[6]. Conversely, the absence of interruptions of chest compressions in studies involving nonasphyxial cardiac arrest improves blood flow and neurologic outcomes in animal studies[7], and survival in human studies[8]. The addition of continuous CC with non-synchronized positive-pressure ventilations might be an adequate strategy. However, when performed by trained healthcare providers in out of hospital cardiac arrest in adults, this approach has not shown to be superior to standard CPR[6]. Brief interruptions (median 2.4, IQR 1.4–7.0 seconds.) in a setting with well-trained rescuers attending in-hospital pediatric cardiac arrest showed no impact on blood pressure before and after the interruption[9]. To our knowledge, there are no human or animal studies comparing synchronized and non-synchronized BVMV in pediatric CPR.

On the other hand, recent guidelines emphasize the importance of high-quality chest compressions for the return of spontaneous circulation (ROSC) during pediatric CPR [4]. Several devices that provide feedback on compressions have been developed to improve the consistency and quality of chest compressions. These devices seem to improve the quality of resuscitation [10–13] as well as the rates of ROSC [14]. As far as we know, there are no studies that have compared the use of these devices regarding the rate of ROSC in children or infant animal models or CPR.

The objective of our study was to compare the effect of synchronized and non-synchronized BVMV with chest compression and between guided and non-guided CC with a real-time feedback-device in a pediatric animal model of asphyxial CA. The secondary aim was to analyze quality of CC during resuscitation and its impact on the return of spontaneous circulation and hemodynamic parameters.

## Methods

We designed a randomized controlled experimental clinical trial. The experiments took place in the Department of Experimental Medicine and Surgery of the Gregorio Marañon University Hospital in Madrid, Spain. The study was approved by the Local Ethics Committee for Animal Research of the Gregorio Marañon University Hospital and was carried out by qualified staff. International guidelines for ethical conduct in the care and use of experimental animals were applied throughout the study.

### Animal model

Sixty holoxenic 3 month old miniature piglets weighing between 7 and 16 kg were used for the study. Cardiac arrest was induced as described in previously published articles from our research group [15–17].

### Animal preparation

The animals were premedicated with intramuscular ketamine (15 mg/kg) and atropine (0.02 mg/kg). An intravenous (iv) line was inserted into a peripheral vein in the ear. Continuous electrocardiography (ECG) and peripheral pulse oximetry (SpO_2_) were monitored. Animals were anesthetized with boluses of propofol (5 mg/kg), fentanyl (5 μg/kg) and atracurium (0.5 mg/kg) for oral endotracheal intubation with a cuffed tracheal tube. Sedation and muscle relaxation were maintained by continuous infusion of propofol (10 mg/kg/h), fentanyl (10 mcg/kg/h) and atracurium (2 mg/kg/h) throughout the procedure.

Animals were mechanically ventilated using volume-controlled ventilation with the following settings: tidal volume 10 ml/kg, respiratory rate 25 bpm, PEEP 4 mmHg, FiO2 30%. Tidal volume was adjusted to maintain arterial pCO2 between 35 and 45 mmHg.

Cannulation of central arterial and venous accesses was ultrasound-guided. A 5.5-French triple-lumen catheter was inserted in the femoral vein for continuous central venous pressure (CVP) monitoring and for fluid and medication infusion. A 4F PiCCO catheter (PiCCO, Pulsion Medical System, Munich, Germany) was placed in the contralateral femoral artery for invasive monitoring of arterial blood pressure, temperature and other hemodynamic parameters as well as for blood sample extraction. An arterial blood flow sensor was surgically placed in the left carotid artery in order to assess cerebral blood flow throughout the experiment (Transonic Systems Inc, Ithaca, New York, USA).

Maintenance fluids containing glucose and saline (40 ml/h) were infused throughout the experiment. A heating blanket was used to maintain normothermia (considering normal temperature limits for pigs). Animals were placed in a v-shaped trough to avoid displacement during CPR.

Pediatric CPR electrodes were applied onto piglets´ back and sternum (in the place of chest compressions) and connected to a Zoll monitor/defibrillator (ZOLL Medical Corporation, Chelmsford, MA, USA).

### Hemodynamic and respiratory parameters

ECG and pulse oximetry were monitored continuously. Central venous and arterial catheters were connected to a PiCCO system for continuous hemodynamic monitoring (heart rate, arterial blood pressure, temperature). Cerebral (ScO_2_) and splanchnic (SsO_2_) oxygen saturations were monitored by near-infrared spectroscopy (NIRS) (INVOS Cerebral Oximeter monitor, Somanetics, Troy, Michigan, USA). A flow monitor (Transonic Systems Inc, Ithaca, New York, USA) connected to the sensor placed in the internal carotid artery offered continuous monitoring of carotid arterial blood flow (CaBF). An S5 monitor (Datex Ohmeda S5, Madison, Wisconsin, USA) measured EtCO2.

Blood gas analyses were processed in a GEMPremier 3001 gas analyzer (Instrumentation Laboratory, Lexington, Kentucky, USA).

### CPR-quality parameters

A Zoll R Series Monitor/Defibrillator recorded CPR-quality parameters. Depth and rate of CC and time without compressions were registered. Compression depth between 3.8 and 5 centimeters (cm) and a rate between 100 and 120 compressions per minute (cpm) were considered optimal.

### Experimental protocol

After a 30-minute stabilization period, baseline data were collected and arterial blood gases were drawn to assess ventilation and oxygenation. A dose of atracurium (1 mg/kg) was administered immediately before extubating the piglets. Cardiac arrest (CA) was defined as a mean arterial pressure (MAP) under 25 mmHg.

CPR was started after at least 10 minutes of asphyxia, when cardiac arrest had been already diagnosed. ROSC was defined as the presence of heart rate higher than 60 bpm and systolic arterial pressure (SAP) higher than 50 mmHg. ROSC was assessed every 3 minutes during CPR, coinciding with the change of rescuer for chest compressions.

The animals were randomized into four groups according to BVMV (synchronized and non-synchronized with CC) and to CC feedback (with and without CC real-time feedback): Group A: guided-CC and synchronized ventilation; group B: guided-CC and non-synchronized ventilation; group C: non-guided CC and synchronized ventilation; and group D: non-guided CC and non-synchronized ventilation.

In accordance with the European Resuscitation Council Guidelines for Resuscitation of 2015, CPR was started with five rescue breaths. Manual CC and bag-valve-mask ventilation (BVMV) were performed by expert pediatric CPR providers.

Synchronized ventilation was delivered at a 15:2 CC to ventilation ratio. When BVMV was not synchronized with CC, ventilations were delivered at a metronome-tailored rate of 30 bpm. according to results of a previous study [15]. The CPR electrodes were applied on all piglets to record the quality of CC. However, the rescuer only received real-time feed-back on CC following the indications of the device screen, in the appointed groups (A and B). Guided-CC were defined as the performance of CC with depth and rate according to the visual real-time feedback of the defibrillator.

The following drugs were administered during CPR: epinephrine 0.02 mg/kg/dose every 3 minutes and sodium bicarbonate (1 mEq/kg/dose) at 9 and 18 minutes of CPR. If a shockable rhythm was detected, animals were defibrillated (4 J/kg) and drug therapy (epinephrine 0.02 mg/kg/dose and amiodarone 5 mg/kg/dose) was administered after the third, fifth and seventh defibrillation if the shockable rhythm persisted (maximum of three doses).

Animals that achieved ROSC were observed for 60 minutes. When the observation period was over, surviving animals were euthanized using a single dose of propofol (10 mg/kg) and potassium chloride (20 mEq/dose).

The following parameters were collected at baseline, 5 minutes after extubation, before starting CPR and every 3 minutes during CPR (during CC and before the assessment of ROSC and change of rescuer for CC): Heart rate and rhythm, systolic arterial pressure (SAP), diastolic arterial pressure (DAP), mean arterial pressure (MAP), SpO_2_, ScO_2_, SsO_2_, CaBF, temperature, etCO2, FiO2 and arterial blood gases. Time from extubation to CA was also registered.

**Fig 1:**
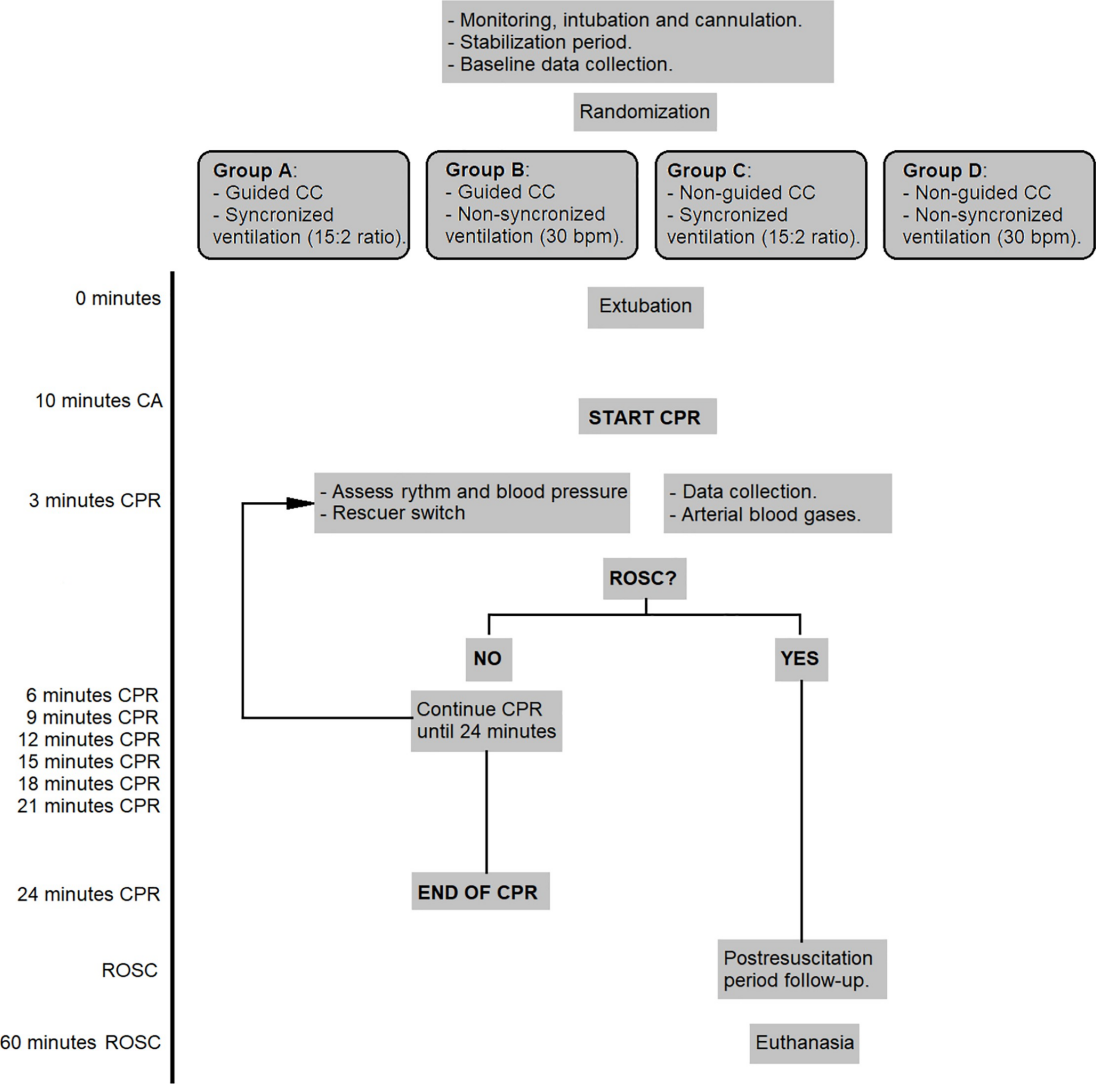
Brief summary of the protocol.

Arterial blood gases were drawn at baseline and after 3, 9, 18 and 24 minutes of CPR. Resuscitation was continued until ROSC or up to a maximum of 24 minutes. Fig 1 shows the algorithm of the experiment.

Broncho-pulmonary hemorrhage was defined as gross bleeding appearing in the airway. An autopsy was performed to all animals with ventilation difficulties during CPR (airway bleeding, insufficient thoracic excursion or chest wall deformity). The presence of bronchopulmonary hemorrhage, hemothorax, cardiac injury, hemopericardium or lung bleeding were considered as severe CPR-related thoracic injuries.

### Statistical analysis

The SPSS statistical package, version 20.0 (SPSS Inc, Chicago, USA) was used for statistical analysis. Normal distribution of variables was tested with the Kolmogorov-Smirnov test. Continuous variables are expressed as medians with interquartile ranges and categorical variables as percentages. Chi-squared (χ2) test was used to compare categorical variables, the Kruskal Wallis and U-Mann Whitney tests for comparing continuous variables, and the Pearson correlation index to assess correlations between continuous variables. P values less than 0.05 were considered significant.

## Results

Sixty piglets were included in the study, 15 in each group. Baseline characteristics are shown in Table 1. There were no significant differences in weight, length, temperature, SAP, DAP, MAP, CVP, heart rate, or cardiac output between groups at baseline.

**Table 1:** Comparison of hemodynamic and respiratory parameters at baseline between groups (median and interquartile range).

	Groups				
Parameters	Group A	Group B	Group C	Group D	p
**Weight (kg)**	12.3 (11.0–12.8)	11.5 (10.8–13.6)	11.8 (11.0–12.7)	12.0 (9.9–13)	0.864
**Length (cm)**	74 (72–78)	75 (71–78)	76 (73–76)	75 (70–76)	0.716
**Heart rate (bpm)**	102(85–110)	93(81–111)	106(92–118)	113(96–125)	0.494
**SAP (mmHg)**	96 (88–104)	99 (91–119)	97 (93–125)	103 (82–119)	0.721
**DAP (mmHg)**	48 (45–55)	51 (43–64)	51 (47–58)	53 (46–62)	0.543
**MAP (mmHg)**	69 (62–74)	67 (62–86)	67 (66–83)	72 (62–85)	0.502
**CVP (mmHg)**	6 (4–11)	7 (3–9)	9 (5–10)	7 (5–12)	0.737
**CaBF (ml/min)**	42 (28–63)	46 (42–52)	42 (37–60)	55 (41–64)	0.849
**ScO**_**2**_ **(%)**	54 (42–67)	58 (49–66)	54 (45–62)	58 (49–72)	0.801
**SsO**_**2**_ **(%)**	56 (51–62)	56 (51–61)	55 (50–62)	60 (56–62)	0.209
**Temperature (°C)**	37.8 (36.8–39)	37.8 (36.6–38.9)	38.7 (37.9–39.5)	38.2 (36.9–39.3)	0.167
**pH**	7.47 (7.46–7.48)	7.47 (7.45–7.51)	7.44 (7.42. 7.48)	7.48 (7.40–7.50)	0.523
**PaCO**_**2**_ **(mmHg)**	43 (40–46)	41 (39–45)	46 (41–48)	42 (41–52)	0.674
**PaO**_**2**_ **(mmHg)**	155 (106–182)	156 (137–185)	153 (118–186)	158 (139–198)	0.900
**SpO**_**2**_ **(%)**	100(95–100)	100 (98–100)	98 (97–100)	100 (99–100)	0.198
**Lactate (mmol/L)**	0.8 (0.6–1.1)	0.8 (0.5–1.1)	0.7 (0.6–1.0)	0.7 (0.5–0.9)	0.543

(CaBF) carotid artery blood flow; (SAP) Systolic arterial pressure, (DAP) diastolic blood pressure, (MAP) mean arterial pressure, (CVP) central venous pressure, (ScO_2_) cerebral oxygen saturations, (SsO_2_) splanchnic oxygen saturation, (SpO_2_) peripheral pulse oximetry.

Mean time from extubation to CA was 7.4 ± 1.2 minutes. Time to CA was longer in survivors (8.3 ± 0.9 minutes) than in non-survivors (6.7 ± 0.8 minutes), p<0.001. No significant differences were found in the time to CA between different CPR-groups (p = 0.626).

Fifty-two piglets (86.6%) had non-shockable rhythms at 10 minutes after asphyxia and only two animals (3.3%) had asystole, with no significant differences between groups (p = 0.395). No differences were found in hemodynamic parameters before beginning CPR between groups: heart rate 72 bpm (60–79.50), p = 0.172, SAP 24 mmHg (16–35.75), p = 0.643, DAP 16.5 mmHg (11–22) p = 0.616 and MAP 15 mmHg (11–22) p = 0.507.

### Return of spontaneous circulation

**Fig 2:**
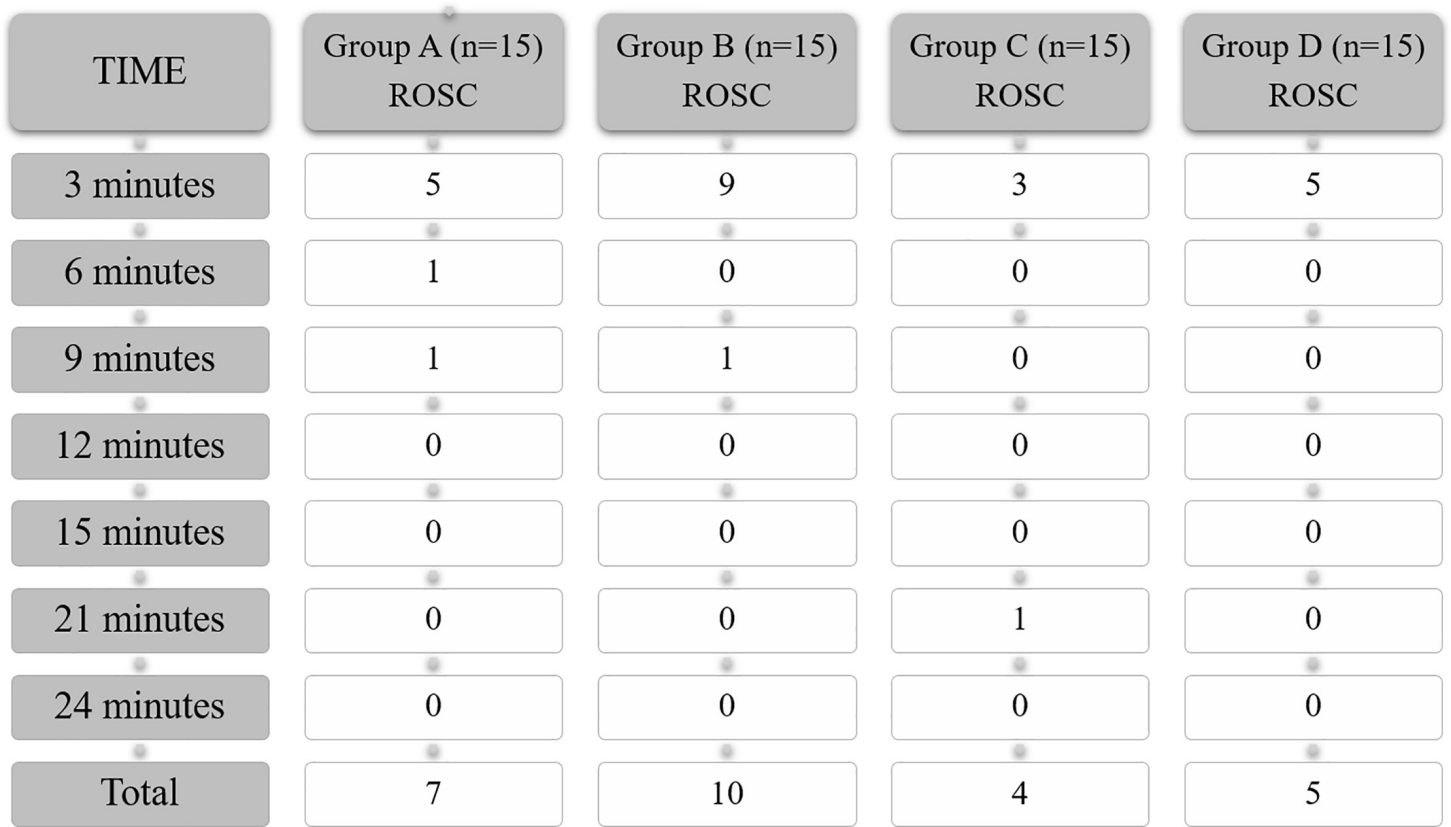
Recovery of spontaneous circulation (ROSC) timeline. Number of piglets that have achieved ROSC at different times of resuscitation. ROSC: Recovery of spontaneous circulation.

ROSC was achieved in twenty-six (46.5%) animals: 7 in group A (46.7%), 10 in group B (66.7%), 4 in group C (26.7%) and 5 in group D (33.3%). Fig 2 shows the evolution of the four groups.

ROSC rates were significantly higher in group B than in all the other groups (66.7% vs 35.6%, p = 0.035). ROSC rates were higher in the groups with guided-CC (A+B: 56.7% vs C+D: 30%; p = 0.037). Non-synchronized ventilation (B+D) also showed a higher but not statistically significant ROSC rate than synchronized ventilation (A+C) (50% vs 36.7%, p = 0.297).

Median resuscitation time until ROSC was 3 minutes (IQR 3–3). Only four animals (15.4%) achieved ROSC after 3 minutes of CPR.

### Hemodynamic parameters

There were no significant differences in hemodynamic parameters between the four groups throughout the resuscitation period. There were only differences in EtCO_2_, but no differences were found in arterial pCO_2_. Table 2 summarizes the hemodynamic parameters at three minutes of resuscitation.

**Table 2:** Comparison of hemodynamic and respiratory parameters at three minutes of CPR (median and interquartile range).

	Groups				
Parameters	Group A	Group B	Group C	Group D	p
**Heart rate (bpm)**	144 (110–170)	138 (115–157)	114 (102–140)	143 (106–181)	0.700
**SAP (mmHg)**	78 (49–140)	119 (66–152)	78 (64–95)	82 (60–108)	0.552
**DAP (mmHg)**	22 (12–102)	42 (18–62)	11 (10–35)	29 (14–42)	0.143
**MAP (mmHg)**	27 (15–46)	55 (27–88)	19 (16–32)	28 (21–59)	0.181
**CVP (mmHg)**	12 (10–16)	13 (10–18)	14 (11–20)	14 (10–18)	0.404
**ScO**_**2**_ **(%)**	30 (15–46)	27 (20–28)	21 (15–36)	45 (30–55)	0.255
**SsO**_**2**_ **(%)**	42 (31–48)	42 (38–50)	31 (25–42)	40 (30–45)	0.277
**CaBF (ml/min)**	3 (0–54)	16 (3–65)	5 (2–9)	14 (4–56)	0.563
**Temperature (°C)**	37.7(37.0–38.7)	37.5 (36.5–38.7)	38.5 (37.6–39.2)	37.8 (38.0–38.6)	0.320
**pH**	7.13 (7.11–7.26)	7.23 (7.12–7.31)	7.14 (7.04–7.27)	7.24 (7.15–7.31)	0.260
**pCO**_**2**_ **(mmHg)**	70 (48–76)	57 (40–69)	74 (49–93)	54 (40–67)	0.119
**pO**_**2**_ **(mmHg)**	66 (41–107)	72 (51–111)	60 (53–121)	66 (54–106)	0.955
**SpO**_**2**_ **(%)**	72 (61–79)	76 (74–91)	87 (74–94)	73 (50–76)	0.180
**EtCO**_**2**_ **(mmHg)**	10 (4–17)	8 (6–14)	8 (5–10)	1 (0–5)	0.023
**Lactate (mmol/L)**	7.3 (6.9–8)	6.9 (6.3–8)	7 (6.5–7.9)	6.9 (6.3–7.2)	0.527

(CaBF) carotid artery blood flow; (SAP) Systolic arterial pressure, (DAP) diastolic blood pressure, (MAP) mean arterial pressure, (CVP) central venous pressure, (ScO_2_) cerebral oxygen saturations, (SsO_2_), splanchnic oxygen saturation, (SpO_2_) peripheral pulse oximetry, (EtCO_2_) end-tidal capnography.

The comparison of hemodynamic parameters after 3 minutes of resuscitation in survivors and non-survivors is shown in Table 3. Animals achieving ROSC after 3 minutes of CPR had higher SAP, MAP, DAP and carotid blood flow than non-survivors.

**Table 3:** Comparison of hemodynamic and respiratory parameters at 3 minutes of cardiopulmonary resuscitation in animals with and without ROSC.

	Return of spontaneous circulation (ROSC)		
Parameters	Non-ROSC (median (IQR))	ROSC (median (IQR))	p
**SAP (mmHg)**	55 (40–70)	116.5 (91.5–162.0)	<0.001
**DAP (mmHg)**	11.5 (10–14)	42 (28.5–96.5)	<0.001
**MAP (mmHg)**	19.5 (15–27)	73 (41.0–128.0)	<0.001
**ScO**_**2**_ **(%)**	21 (15–36)	41.5 (21.5–51.5)	0.061
**SsO**_**2**_ **(%)**	42 (30–45)	40 (31.0–48.0)	0.970
**CaBF (ml/min)**	3 (1–8)	56 (15.0–80.0)	<0.001
**ArterialpH**	7.18 (7.10–7.31)	7.16 (7.12–7.28)	0.878
**Arterial pCO**_**2**_ **(mmHg)**	61 (44.0–77.5)	65 (47.0–73.0)	0.811
**Arterial pO**_**2**_ **(mmHg)**	64.5 (42.0–91.0)	75 (53.0–158.0)	0.600
**Arterial lactate**	7.7 (6.6–8.1)	6.9 (6.3–7.2)	0.022

(IQR) interquartile range, (CaBF) carotid arterial blood flow; (SAP) Systolic arterial pressure, (DAP) diastolic blood pressure, (MAP) mean arterial pressure, (ScO_2_) cerebral oxygen saturations, (SsO_2_) splanchnic oxygen saturation.

**Fig 3:**
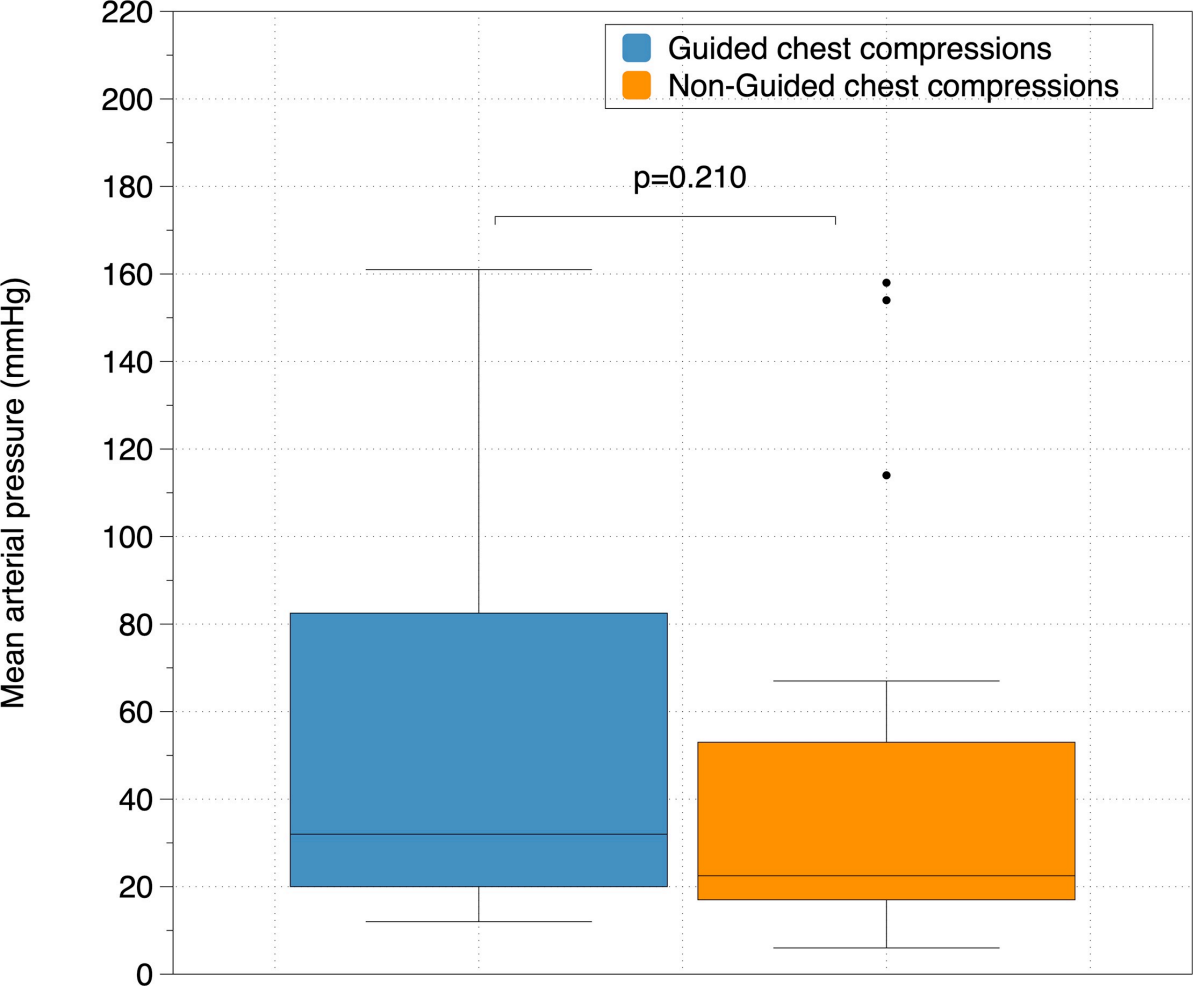
Comparison of Mean Arterial Pressure (mmHg) between guided and non-guided chest compressions.

**Fig 4:**
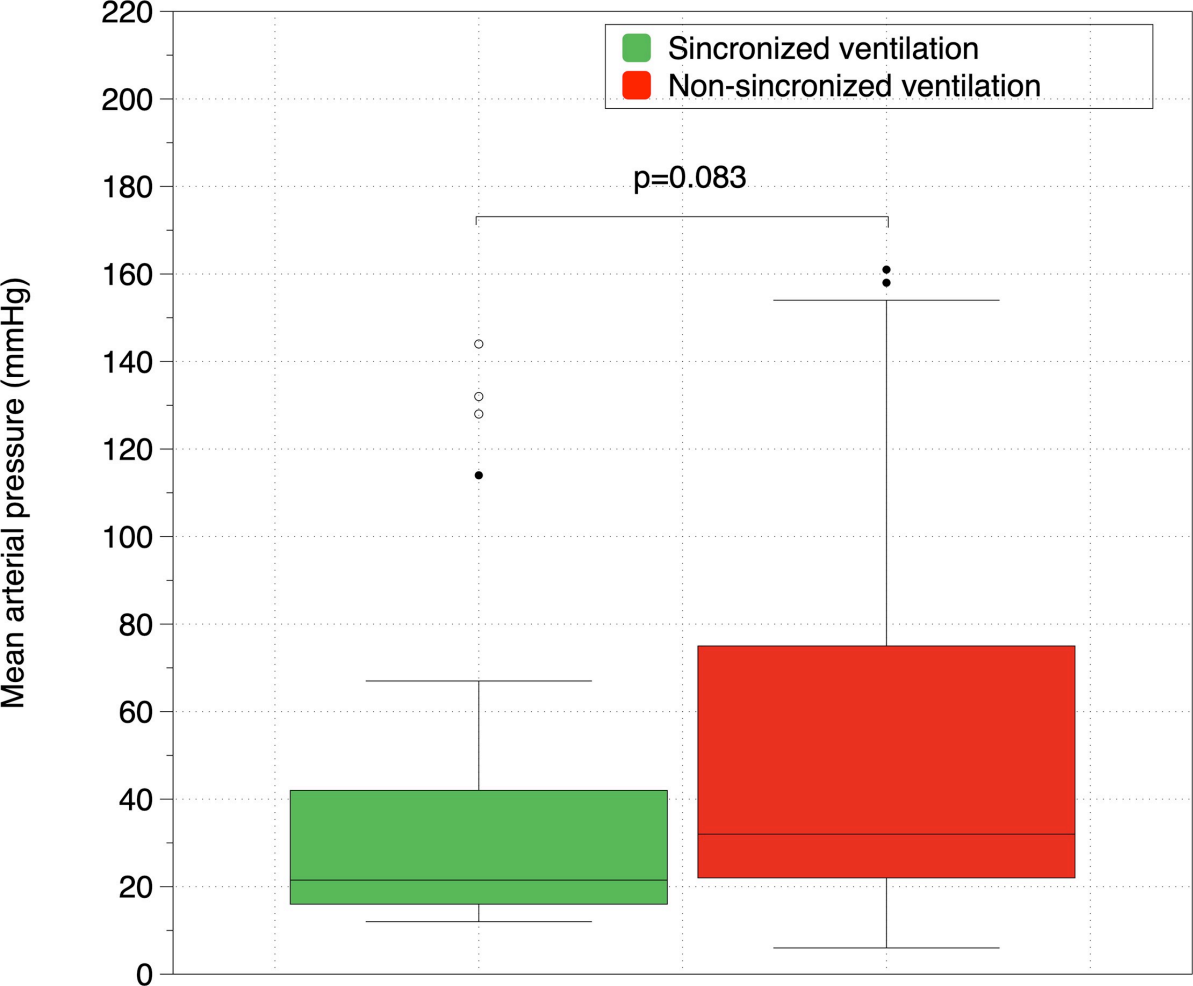
Comparison of Mean Arterial Pressure (mmHg) between synchronized and non- synchronized ventilation.

**Fig 5:**
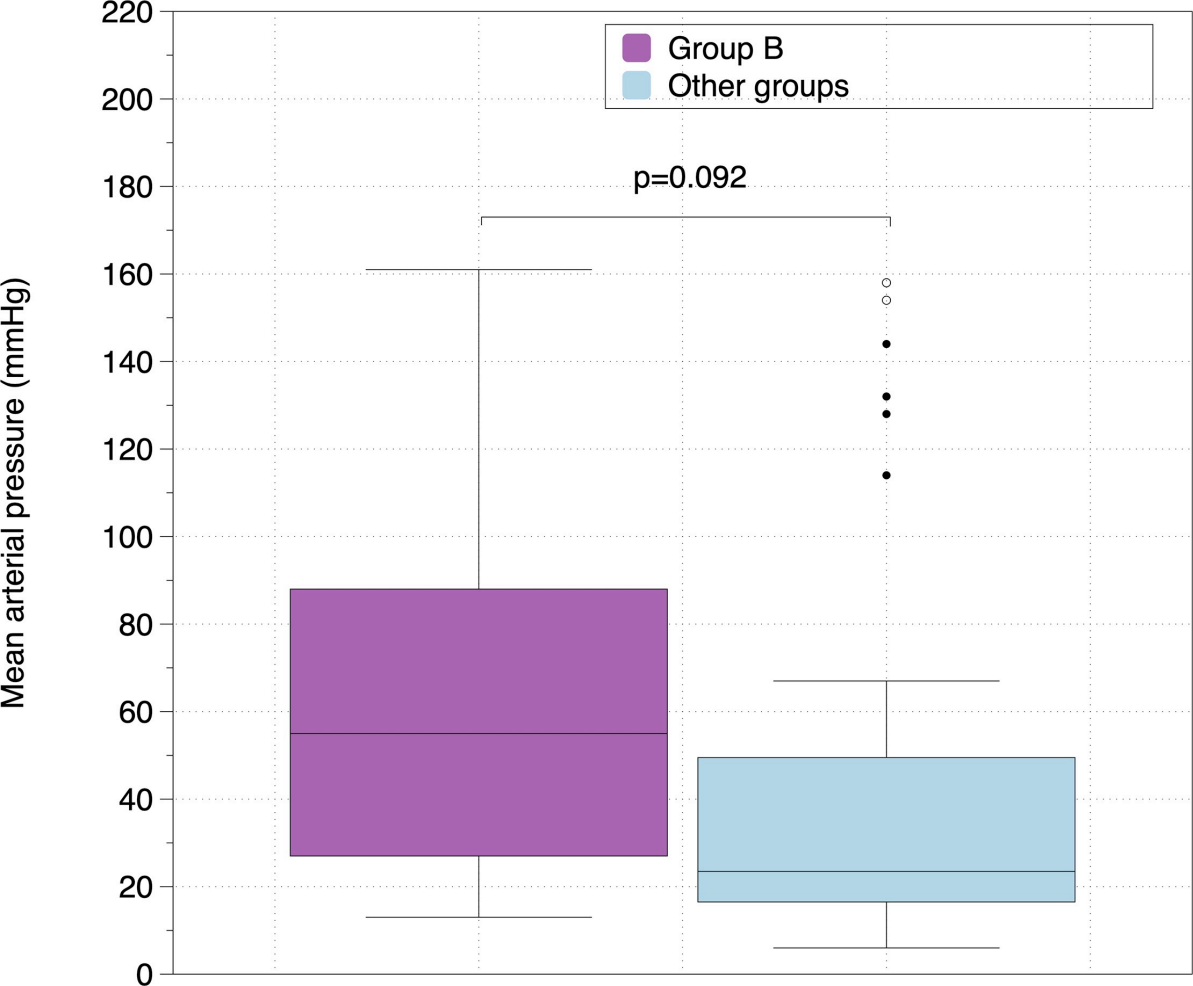
Comparison of Mean Arterial Pressure (mmHg) between group B (guided chest compressions and non-synchronized ventilation) and other groups.

Fig 3 shows the differences in MAP after 3 minutes of CPR between guided and non-guided CC, Fig 4 between synchronized and non-synchronized ventilation and Fig 5 between group B and the rest of groups. MAP was higher in guided CC groups, in non-synchronized BVMV and in group B, but differences did not reach statistical significance. No differences were found in any other hemodynamic parameters.

### Respiratory parameters

There were no significant differences in respiratory parameters between the four groups throughout the experiment. Table 3 shows respiratory parameters at 3 minutes of CPR in piglets that did and did not achieve ROSC. Piglets with non-synchronized ventilation (groups B and D) showed lower arterial pCO_2_ than those with synchronized ventilation (groups A and C): 57 mmHg (IQR 40–68) vs 71 mmHg (IQR 49–80), p = 0.019. There was a tendency to better arterial pH in piglets receiving non-synchronized ventilation (7.23 (IQR 7.13–7.31) vs 7.14 (IQR 7.07–7.26), p = 0.06), but statistical significance was not achieved. No differences were found in arterial pO_2_ (66 mmHg (51–106) vs 65 mmHg (50–107), p = 0.58).

### Cerebral blood flow and perfusion

There were no differences in CaBF, ScO_2_ and SsO_2_ between the four groups (Table 2) or when comparing guided and non-guided CC or synchronized and non-synchronized BVMV.

### CPR-quality

**Fig 6:**
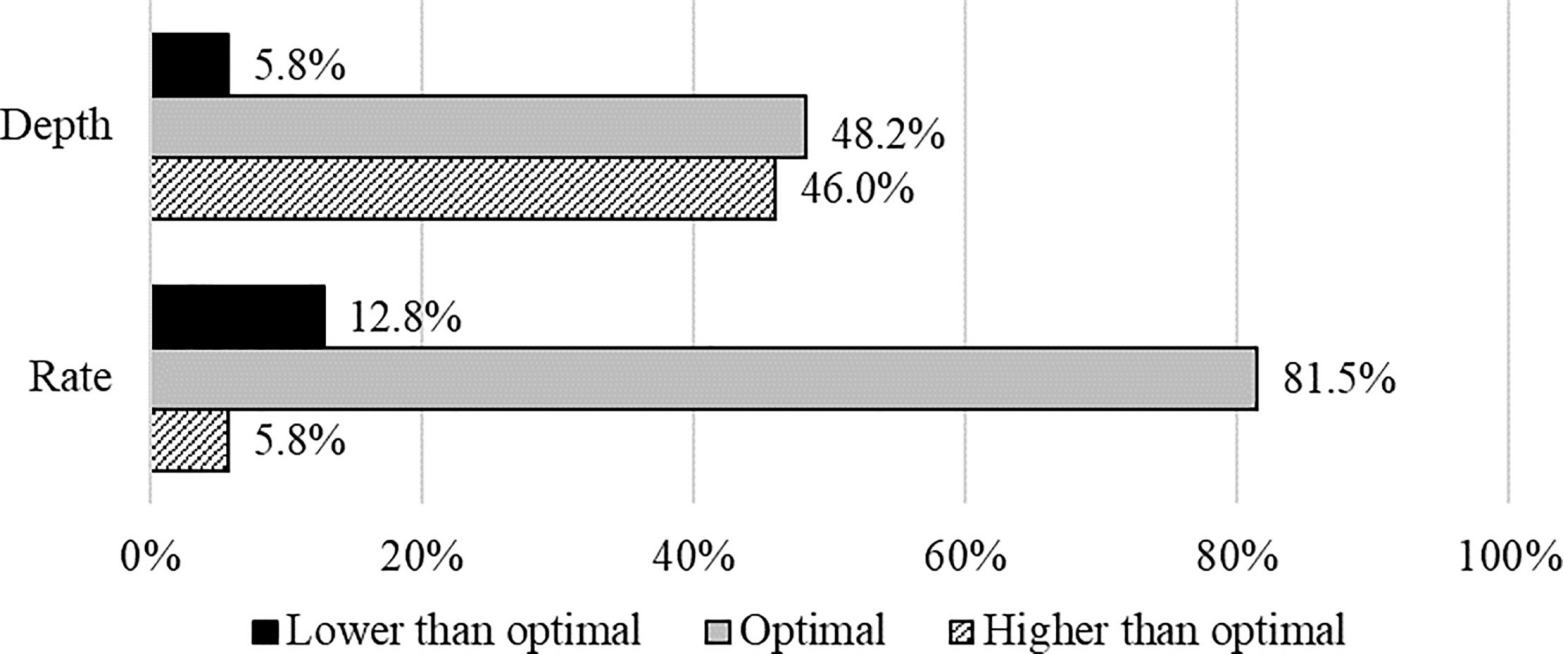
Percentage of chest compressions with optimal rate and depth.

We were able to analyze CPR-quality in 36 animals. In the remaining 24 animals, data analysis was not possible due to loss or to overwriting of new data over the old data in the memory card. Fig 6 shows the percentage of optimal and suboptimal CC in depth and rate. Only 40.0 ± 26.4% of overall CC were optimal in rate and depth. Table 4 summarizes the differences in CC-quality between groups, showing statistically significant differences in rate but not in depth.

**Table 4:** Comparison of chest compression quality between groups.

	Groups				
**Chest compressions quality**	**Group A (**Median (IQR))	**Group B (**Median (IQR))	**Group C** **(**Median (IQR))	**Group D** **(**Median (IQR))	p
**Number of animals**	8	10	9	9	
**Depth (cm)**	5.1 (4.7–5.4)	4.9 (4.7–5.0)	5.5 (5.0–5.9)	4.8 (4.6–5.2)	0.140
**Rate (cpm)**	101.0 (98.5–102.5)	106.1 (102.6–109.5)	101.9 (100. -105.2)	107.3 (106.2–111.2)	0.021

(cm) centimeters; (cpm) compressions per minute; (IQR) interquartile range.

Piglets achieving ROSC received shallower CC than non-survivors (4.7 cm (IQR 4.5–5.2) vs 5.1 cm (IQR 4.9–5.4), p = 0.047) with no significant differences in CC rate (106 cpm (101–109) vs 103 cpm (100–106); p = 0.226). The percentage of optimal compressions (in depth and rate) was higher in animals that achieved ROSC than in those who did not, although these results did not reach statistical significance (48.3% vs 31.8% p = 0.062).

No differences were found in depth or rate of CC between guided and non-guided CC groups (4.9 cm (IQR 4.7–5.2) vs 5.1 cm (IQR 4.6–5.5); p = 0,606 and 103 cpm (IQR 101–109) vs 106 (104–111); p = 0.563, respectively). The depth of CC during the first 3 minutes of resuscitation was similar in synchronized and non-synchronized ventilation groups (4.9 cm (IQR 4.5–5.7) vs 4.9 cm (IQR 4.5–5.3), p = 0.30). However, when considering total resuscitation time, CC were deeper in the groups with synchronized than in those with non-synchronized ventilation (5.3 cm (IQR 4.7–5.6) vs 4.9 cm (IQR 4.6–5.1), p = 0.038).

Time without chest compressions (hands-off time) was significantly shorter in piglets with non-synchronized than in those with synchronized ventilation (0.02 minutes (0.02–0.8) vs 3.4 minutes (0.7–5.3), p<0.001) and in those that achieved ROSC over those who did not (0.02 minutes (0.02–0.53) vs 2.35 (0.82–5.31), p<0.001).

Hemodynamic parameters such as MAP did not show a significant correlation with the depth of CC in the first 3 minutes of CPR (r = -0.32; p = 0.058). However, a negative correlation was found between hands-off time and MAP (r = - 0.35; p 0.038).

### Side effects

Sixteen piglets underwent autopsy, but only 6 of them (10%) revealed severe injuries (3 haemothorax, 1 hemopericardium, 2 right atrium tear). Only two of these six piglets with severe injuries had clinical broncho-pulmonary hemorrhage. On the other hand, ten (16.6%) animals had clinically evident broncho-pulmonary hemorrhage. Altogether severe CPR-related injuries were observed in 14 piglets (23.3%).

Piglets who suffered severe injuries had received deeper CC (5.4 cm (5.2–5.5) vs 4.9 cm (4.6–5.2), p = 0.017). None of the 14 animals that suffered severe CPR-related injuries survived. None of the piglets that achieved ROSC presented severe injuries, as opposed to the piglets that never achieved ROSC, which presented them in 41.2% of the cases (p<0.01).

The incidence of severe CPR-related injuries did not show any significant differences between groups (5 in group A (33.3%), 2 in group B (13.3%), 3 in group C (20%) and 4 in group D (26.7%), p = 0.602), nor was it related to the type of ventilation (20% in non-synchronized vs 26.7% in synchronized ventilation groups, p = 0.542).

## Discussion

Cardiac arrest has a high mortality rate in children. Early cardiopulmonary resuscitation and appropriate CPR maneuvers are crucial to improve survival and reduce neurological sequelae. The first minutes of resuscitation are essential because, as the time of CPR increases, the chances of survival decrease [3,4].

Quality chest compressions, adequate ventilation, and a well-coordinated resuscitation minimizing disruptions are the essential steps in advanced CPR in children. This present study addresses the influence of several of these factors on the return of spontaneous circulation. Furthermore, it describes the evolution of hemodynamic variables, ventilation parameters and cerebral perfusion in a pediatric animal model of CA. This study reveals some novel and relevant data on pediatric CPR, which could be useful for clinical studies in children.

### Guided versus non-guided chest compressions

New devices that provide real-time feedback on CC are intended to optimize chest compressions [14,18,19]. Their effectiveness has been tested in different simulation studies [20,21] but, to date, only one clinical study in adult patients with CA finds these devices to improve survival [14].

Our study is, to our knowledge, the first to describe that guided CC is associated with significantly higher ROSC rates than non-guided resuscitation in a pediatric animal model. Even though guided CC were associated with increased ROSC and a tendency to slightly higher MAP (statistically not significant), no significant differences were found in depth or rate of CC between guided and non-guided CC.

According to our results, the use of these real-time feedback devices could improve survival in pediatric CPR. Clinical studies in children are needed to confirm their usefulness and effectiveness during CPR.

### Synchronized versus non-synchronized ventilation

Although endotracheal intubation is the best method to secure the airway and to ventilate the patient, airway management during cardiopulmonary arrest is a contentious issue and there is still much controversy about when is the best moment for intubation and whether or not it is always necessary during pediatric CPR [22,23].

Experts recommend bag mask ventilation for initial airway management in advanced CPR. Current guidelines recommend that ventilation with BVMV be synchronized with chest compressions at a ratio of 30:2 in adults and 15:2 in children.

Synchronized ventilation allows, in theory, better ventilation as chest compressions do not interfere with the delivered breaths, but it increases the time without chest compressions. Nichol et al [24] compared continuous CC vs synchronized CC in adult out-of-hospital CA with a 30:2 ventilation to CC ratio, and did not find any differences in survival or neurologic outcomes.

Although ventilation is an important matter in pediatric CA, there is still great variability in terms of airway management according to the results of a broad multinational survey including 46 different countries [25]. However, there are no published studies in children or pediatric animal models analyzing the effectiveness of BVMV while delivering continuous chest compressions. Our study shows that non-synchronized ventilation provided better ventilation with lower arterial pCO2 and higher pH than synchronized ventilation. After 3 minutes of resuscitation, arterial pCO2 values were not in the hyperventilation range despite using higher respiratory rates than what international guidelines recommend. The use of higher respiratory rates is based in the results of previous experimental studies from our research group using the same pediatric model of asphyxial CA, in which higher respiratory rates showed a tendency towards greater ROSC rates [15].

Yang et al (15) compared synchronized and non-synchronized ventilation in an adult animal study and did not find any differences in gas exchange. Nevertheless, there are some major differences between this study and ours. In the first place, it is an adult (not pediatric) animal model. Secondly, cardiac arrest was provoked by inducing ventricular fibrillation instead of using an asphyxial model of CA. In the third place, synchronized ventilation was delivered with a ratio of 30:2 instead of 15:2 and the non-synchronized ventilation at a rate of 10 breaths per minute rather than 30 bpm. All these differences could explain our differing results, as the main cause of CA in children is asphyxia leading to hypoxia and ischemia and, in consequence, ventilation during CPR plays a greater role in pediatric than in adult CA.

Many studies in adult CA describe the deleterious effects of hyperventilation during CPR in hemodynamic performance and, ultimately, in ROSC[26–28], which is why current guidelines recommend a respiratory rate of 10–12 bpm. Nevertheless, our study did not show any significant differences in MAP, arterial carotid blood flow or cerebral saturation between synchronized and non-synchronized ventilation groups, even considering that non-synchronized groups received 30 bpm.

In our study, non-synchronized ventilation improved arterial pCO_2_ but not arterial pO_2_. Kill et al [29,30] designed a ventilator model called Chest Compression Synchronized Ventilation that insufflates a short positive pressure ventilation exactly at the start of each chest compression. They compared this ventilation modality with intermittent positive pressure ventilation (IPPV) in a pig model of CA. Their novel ventilation modality achieved higher arterial pO2 than IPPV and avoided an arterial blood pressure drop during ventilation.

We did not find any significant differences in the depth of CC in the first 3 minutes of resuscitation when comparing synchronized and non-synchronized ventilation. However, when considering total resuscitation time, CC were deeper in the groups with synchronized than in those with non-synchronized ventilation. This could be due to fatigue of the rescuer, which is probably greater when CC are continuous. Even so, the depth of CC in non-synchronized ventilation groups was maintained within the recommended depth range and we must also consider that deeper CC were associated with a higher incidence of severe CPR-related injuries.

Moreover, non-synchronized ventilation significantly decreased the time without compressions, which has been directly related to increased survival rates [3,4].

On the other hand, in the study mentioned above by Yang et al [31] in an adult animal model, they describe a greater incidence of CPR-related injuries (rib fractures) in the non-synchronized ventilation group of pigs. In our study, however, although the incidence of severe injuries is high, non-synchronized ventilation was not associated with a higher incidence of this.

This difference is probably due to the way CC were delivered: we delivered manual CC whereas Yang et al used a mechanical thoracic compression device. We report a greater incidence of broncho-pulmonary hemorrhage when using mechanical chest compression devices in a previous study in piglets [12].

Finally, there is also a tendency for non-synchronized ventilation to increase ROSC (50% versus 36.7%, p = 0.297), although differences did not reach statistical significance (probably because of the small sample size).

Therefore, according to our results, non-synchronized ventilation with a higher-than-recommended ventilation rate (30 bmp) achieved significantly better ventilation parameters without hyperventilated animals and reduced the time without compressions without any deleterious effects on hemodynamics, cerebral perfusion, or CPR-related injuries.

### Guided chest compressions and non-synchronized ventilation

Our study shows that the combination of guided CC and non-synchronized ventilation obtained significantly higher ROSC rates than all the other groups. Nevertheless, our results did not reveal any significant differences in the quality of CC (depth or rate), in hemodynamic variables or in any ventilation parameters between this group of piglets and the rest.

### Limitations

Our study has several limitations. In the first place, this validated animal model attempts to reproduce pediatric asphyxial cardiac arrest. It is obvious that results from experimental animal models must be interpreted with caution and cannot be directly extrapolated to humans. Anatomic differences between piglets and humans could potentially play an important role in two major aspects: airway (affecting the effectiveness of BVMV) and thorax (piglets have a keel-shaped thorax, which could affect the effectiveness of CC in terms of hemodynamic effects and CPR-related injuries).

Secondly, a substantial amount of data on CPR-quality was lost due to technical problems. Therefore, the number of animals in each group could be insufficient to achieve statistical significance in some of the analyses.

On the other hand, blood pressure values at three minutes could reflect not only the efficacy of CC, but the spontaneous cardiac activity in animals that had recovered spontaneous circulation. We thought of registering BP values before ROSC and during CC, but, as it is not possible to predict the moment at which ROSC will occur, BP values were measured at the same time in all the animals in order to standardize the groups. Furthermore, tidal volume and peak pressure were not measured and for this reason we could not evaluate their effect on ventilation and the evolution of resuscitation.

## Conclusions

This pediatric animal model of asphyxial CA shows that non-synchronized ventilation and guided CC obtained significantly higher ROSC rates than the other modalities of resuscitation. Guided-CC achieved higher rates of ROSC than non-guided CC. Non-synchronized ventilation was associated with better ventilation parameters and less time without CC, with no differences in hemodynamic parameters, cerebral blood flow or in the incidence of severe injuries. Moreover, there was a correlation between time without CC and mean arterial pressure. Deeper CC were associated with severe injuries and lower ROSC rates. Nevertheless, studies in children are necessary to confirm our results.

## Supporting information

S1 Project Database CPR pig model data. (SAV)
